# Metabolomics for early-stage lung adenocarcinoma: diagnostic biomarker screening

**DOI:** 10.3389/fonc.2025.1535525

**Published:** 2025-03-11

**Authors:** Danxiong Sun, Yanhong Du, Rufang Li, Yunhui Zhang

**Affiliations:** ^1^ Faculty of Life Science and Technology, Kunming University of Science and Technology, Kunming, China; ^2^ Department of Pulmonary and Critical Care Medicine, The First People’s Hospital of Yunnan Province, The Affiliated Hospital of Kunming University of Science and Technology, Kunming, China

**Keywords:** early-stage lung adenocarcinoma, metabolomics, biomarker, diagnostic model, liquid chromatography-mass spectrometry

## Abstract

**Objective:**

This study aimed to identify specific metabolic markers in the blood that can diagnose early-stage lung adenocarcinoma.

**Methods:**

An untargeted metabolomics study was performed, and the participants were divided into four groups: early-stage lung adenocarcinoma group (E-LUAD; n = 21), healthy control group (HC, n = 17), non-cancerous lung disease group (NCC; n = 17), and advanced lung adenocarcinoma group (A-LUAD; n = 25). Plasma metabolite levels that differed in the E-LUAD group compared to the other three groups were identified via liquid chromatography–mass spectrometry (LC–MS). Principal component analysis (PCA) and partial least squares discriminant analysis (PLS-DA) were performed at metaX for statistical analysis. A Venn diagram was constructed to identify overlapping differential metabolites of the class comparisons. The data were randomly divided into a training set and a validation set. Based on the overlapping differential metabolites, the diagnostic model was constructed. The discrimination of the model was evaluated using the area under the curve (AUC).

**Results:**

A total of 527 metabolites were tentatively identified in positive ion mode and 286 metabolites in negative ion mode. Compared with the HC group, 121 differential metabolites were identified. Compared with the NCC group, 67 differential metabolites were identified. Compared with the A-LUAD group, 54 differential metabolites were identified. The Venn diagram showed that 29 metabolites can distinguish E-LUAD from HC and NCC and that four metabolites can distinguish E-LUAD from HC, NCC, and A-LUAD. The feature metabolites were selected to establish the diagnostic model for E-LUAD. The AUC value of the training set was 0.918, and it was 0.983 in the validation set.

**Conclusion:**

Blood metabolomics has potential diagnostic value for E-LUAD. More medical studies are needed to verify whether the metabolic markers identified in the current research can be applied in clinical practice.

## Introduction

Lung cancer, due to its insidious nature, remains a leading cause of cancer-related deaths worldwide. Adenocarcinoma is now the main histologic type of lung cancer, accounting for almost one-half of all cases (1). Lung adenocarcinoma (LUAD) leads to the majority of deaths attributable to lung cancer. Nowadays, the main technical means for lung cancer screening is low-dose computed tomography (LDCT). However, the high rate of false positives and misdiagnosis reduces the effectiveness of LDCT (2). It has been proved that early diagnosis can improve the survival rate of lung cancer patients. In addition, LDCT still has potential carcinogenic risks due to radiation exposure. Identifying sensitive and specific early diagnostic biomarkers for lung cancer is currently an urgent issue that needs to be addressed. The development of metabolomics provides strong technical support for screening diagnostic biomarkers.

In recent decades, metabolomics has become an important tool for discovering diagnostic biomarkers of lung cancer, and an increasing number of omics studies have identified many potential biomarkers for lung cancer (3). However, these studies still have certain limitations. First, many studies lack a non-cancerous lung disease group, which raises the possibility that one or more identified biomarkers may be diagnostic of lung pathology without necessarily being specific to lung cancer. Compared to those in healthy individuals, up- or downregulated metabolites in lung cancer patients could be a consequence of secondary changes in lung function that could also occur in benign lung diseases, in which case the biomarkers would not be lung cancer-specific. Second, few studies have focused on the diagnosis of early-stage lung cancer, which is the most technically challenging (4).

In this untargeted metabolomics study, we divided participants into four groups: early-stage lung adenocarcinoma group (E-LUAD), healthy control group (HC), non-cancerous lung disease group (NCC), and advanced lung adenocarcinoma group (A-LUAD). We compared the E-LUAD group with the other three groups, analyzed differential metabolites, and explored diagnostic metabolic biomarkers specific to E-LUAD.

## Participants and methods

### Participants

The blood samples were collected from the First People’s Hospital of Yunnan Province from January to June 2023. The diagnosis of all patients was confirmed. Lung adenocarcinoma was diagnosed through histopathology. All patients with lung adenocarcinoma underwent contrast-enhanced computed tomography (CT) scans of the brain, chest, and abdomen, with female patients also receiving contrast-enhanced CT scans of the pelvis. All lung adenocarcinoma patients underwent single-photon emission computed tomography (SPECT) bone imaging. The TNM staging of lung cancer was determined according to the 8th edition of the TNM classification issued by the International Association for the Study of Lung Cancer (IASLC). Patients with stage TNM I and stage TNM II underwent video-assisted thoracoscopic surgery (VATS), with postoperative histopathology confirming adenocarcinoma. Patients with stage TNM III and stage TNM IV underwent tissue biopsy (with the exception of five cases who underwent medical thoracoscopic pleural biopsy, the rest underwent transbronchial lung biopsy and endobronchial ultrasound-guided transbronchial needle aspiration), with histopathology confirming adenocarcinoma. Pulmonary cryptococcosis, pulmonary sclerosing pneumocytoma, hamartoma, organizing pneumonia, and chronic suppurative inflammation were all diagnosed through surgical lung biopsy. Pulmonary aspergillosis was diagnosed through tissue samples obtained via transbronchial lung biopsy. The remaining non-cancerous lung diseases were diagnosed clinically. Stage TNM I and stage TNM II were set as early-stage lung cancer, and stage TNM III and stage TNM IV as advanced-stage lung cancer.

### Sample collection and preparation

The blood samples of all patients were taken before treatment, invasive medical tests, and surgical intervention. To avoid the effects of food, all of the samples were taken in the morning before breakfast (fasting for at least 8 hours). EDTA anticoagulation tubes were used to collect blood. The collected blood was immediately sent to the laboratory and centrifuged at 4°C and 3,000 *g* for 10 min. The separated plasma was stored in a −80°C refrigerator.

The samples (100 μL plasma) were resuspended with 400 μL prechilled 80% methanol. The mixtures were incubated on ice for 5 min and then centrifuged at 4°C and 15,000 *g* for 20 min. The supernatant was collected and diluted with liquid chromatography–mass spectrometry (LC–MS)-grade water to a methanol concentration of 53%. The samples were centrifuged at 4°C and 15,000 *g* for 20 min. Finally, the supernatant was injected into the LC–MS system analysis. A quality control (QC) sample used for all LC–MS runs was prepared by combining equal volumes of diluted plasma from each participant.

### Instrumental test

An Orbitrap Q Exactive™ HF-X mass spectrometer (Thermo Fisher, Bremen, Germany) coupled with a Vanquish UHPLC system (Thermo Fisher, Germany) was used to perform LC–MS analyses. The mass spectrometer was operated in positive and negative polarity modes. The m/z acquisition range was 100–1,500. Samples were injected onto a Hypersil Gold column (100 × 2.1 mm, 1.9 μm) using a 12-min linear gradient at a flow rate of 0.2 mL/min. The eluents for the positive polarity mode were eluent A (0.1% formic acid (FA) in water) and eluent B (methanol). The eluents for the negative polarity mode were eluent A (5 mM ammonium acetate, pH 9.0) and eluent B (methanol). The solvent gradient was set as follows: 2% B, 1.5 min; 2%–85% B, 3 min; 85%–100% B, 10 min; 100%–2% B, 10.1 min; and 2% B, 12 min. The parameter settings for the mass spectrometer were as follows: MS/MS secondary scanning, data-dependent scans; aux gas heater temperature, 350°C; spray voltage, 3.5 kV; aux gas flow rate, 10 L/min; S-lens radio frequency (RF) level, 60; sheath gas flow rate, 35 psi; and capillary temperature, 320°C.

### Data processing and metabolite identification

To perform peak picking, peak alignment, and quantitation for each metabolite, Compound Discoverer 3.3 (CD3.3; Thermo Fisher) was used to process the raw data files generated by LC–MS. The main parameters were set as follows: signal intensity tolerance, 30%; peak area was corrected with the first QC; actual mass tolerance, 5 ppm; and minimum intensity.

Peak intensities were normalized to the total spectral intensity, and then the molecular formula with the normalized data was predicted. To obtain accurate qualitative and relative quantitative results, the peaks were matched with the mzVault, mzCloud (https://www.mzcloud.org/), and MassList database.

Statistical analyses were performed using the software R (R version R-3.4.3), CentOS (CentOS release 6.6), and Python (Python 2.7.6 version). Data that were not normally distributed were standardized using the following equation to obtain relative peak areas:

rPA $=\frac{rQV}{QVs/QVqc}.$ rPA is the relative peak area. rQV is the sample raw quantitation value, which refers to the result obtained using the CD3.3 software to perform peak integration and calculate the area under the curve for the mass spectrometry peaks corresponding to each metabolite. QVs is the sum of sample metabolite quantitation value. QVqc is the sum of the QC1 sample metabolite quantitation value.

Only the compounds whose coefficient of variation (CV) of relative peak areas in QC samples was less than or equal to 30% were retained, and the metabolite identification and relative quantification results were finally obtained.

### Construction and verification of the diagnostic model

E-LUAD was compared with the other three groups to identify significantly different plasma metabolite levels. A Venn diagram was constructed to identify overlapping differential metabolites of the class comparisons. The data were randomly divided into a training set and a validation set. The overlapping differential metabolites were subjected to logistic regression analysis; then, the diagnostic model was constructed. The receiver operating characteristic (ROC) curve was plotted to evaluate the diagnostic performance of the model. The area under the ROC curve (AUC) was used to evaluate diagnostic accuracy: high accuracy (0.9 < AUC < 1) and moderate accuracy (0.7 < AUC ≤ 0.9). An AUC metabolite score meant the diagnostic accuracy for E-LUAD.

### Statistical analysis

The metabolites were annotated using the Human Metabolome Database (HMDB) database (https://hmdb.ca/metabolites), Kyoto Encyclopedia of Genes and Genomes (KEGG) database (https://www.genome.jp/kegg/pathway.html), and LIPIDMaps database (http://www.lipidmaps.org/). The metabolites with variable importance in the projection (VIP) score >1.0, fold change (FC) > 1.5 or FC < 0.667, and p-value < 0.05 were considered to be differential metabolites. Partial least squares discriminant analysis (PLS-DA) and principal component analysis (PCA) were performed at metaX. The goodness of fit for the PLS-DA models was evaluated using three quantitative parameters: R^2^X, R^2^Y, and Q^2^. Univariate analysis (t-test) was applied to calculate the statistical significance (p-value).

For clustering heatmaps, z-scores of the intensity areas of differential metabolites were used to normalize the data. The functions of metabolites and metabolic pathways were studied using the KEGG database. When the ratio was satisfied by x/n > y/N, the metabolic pathway was considered enriched (x, the number of differential metabolites associated with a specific metabolic pathway; y, the total number of all metabolites associated with a specific metabolic pathway; and n, the number of differential metabolites annotated by KEGG). The metabolic pathway whose p < 0.05 was considered statistically significantly enriched.

GraphPad Prism 8.0 was used for the statistical analysis of baseline data from the four groups of participants. The Kruskal–Wallis statistic was used for the comparison of non-normally distributed data. The chi-square test was used for the statistical analysis of counting data. p-Value less than 0.05 was considered statistically significant.

## Results

### Comparison of general characteristics

The study included four groups: E-LUAD (n = 21), HC (n = 17), NCC (n = 17), and A-LUAD (n = 25).

The NCC group included eight cases of pulmonary infectious diseases (five cases of bacterial pneumonia, one case of active pulmonary tuberculosis, one case of invasive pulmonary aspergillosis, and one case of pulmonary cryptococcosis), three cases of pulmonary sclerosing pneumocytoma, two cases of hamartoma, one case of chronic suppurative inflammation, one case of chronic obstructive pulmonary disease, one case of organic pneumonia, and one case of bronchiectasis.

There were no significant differences in age, smoking status, tumor history, or comorbidity among the four groups (p > 0.05); see **Table 1** for details.

**Table 1:** Baseline characteristics of participants.

N (%)	E-LUAD	HC	NCC	A-LUAD	p-Value
**No.**	21	17	17	25	
Age					0.3914
Age (IQR)	60.00 (50.00, 68.00)	56.00 (47.50, 61.50)	55.00 (46.00, 61.00)	59.00 (50.00, 68.00)	
Range	39–83	39–69	32–78	33–76	
Sex					
Male	8 (38.10)	5 (29.41)	7 (41.18)	11 (44)	0.8107
Female	13 (61.90)	12 (70.59)	10 (58.82)	14 (56)	
Smoking status					0.9395
Never	13 (61.90)	12 (70.59)	12 (70.59)	16 (64)	
Former	2 (9.52)	2 (11.76)	1 (5.88)	4 (16)	
Current	6 (28.57)	3 (17.65)	4 (23.53)	5 (20)	
Tumor history					0.3033
Yes	3 (14.29)	0	1 (5.88)	4 (16)	
No	18 (85.71)	17 (100)	16 (94.12)	21 (84)	
Comorbidity					0.1794
Yes	5 (23.81)	0	2 (11.76)	3 (12)	
No	16 (76.19)	17 (100)	15 (88.24)	22 (88)	
Cancer stage					
I	15 (71.43)				
II	6 (28.57)				
III				6 (24)	
IV				19 (76)	

E-LUAD, early-stage lung adenocarcinoma; HC, healthy control; NCC, non-cancerous lung disease; A-LUAD, advanced lung adenocarcinoma; IQR, interquartile range.

### Potential biomarker identification

A total of 527 metabolites were tentatively identified in positive ion mode (**Supplementary Table 1**) and 286 metabolites in negative ion mode **(Supplementary Table 2**).

### Quality control

Based on the relative quantification values of metabolites, Pearson’s correlation coefficient between QC samples was calculated. All R^2^ values were greater than 0.98 in positive ion mode, and all R^2^ values were greater than or equal to 0.99 in negative ion mode (**Supplementary Figure 1**).

**Figure 1:**
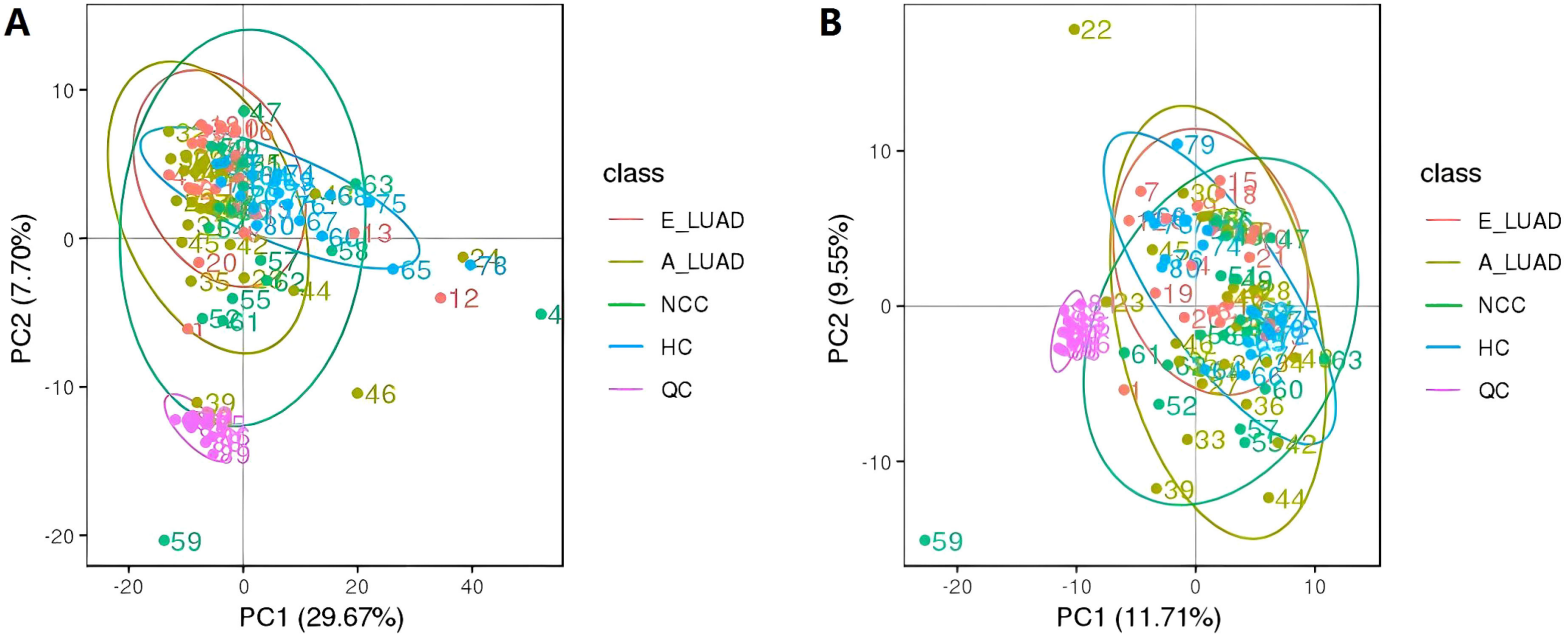
The PCA score plots of all identified metabolites and QC samples. **(A)** The positive ion mode. **(B)** The negative ion mode. PCA, principal component analysis; QC, quality control.

We performed PCA on the peaks extracted from all experimental and QC samples. The PCA score plots (**Figures 1A, B**) demonstrated good clustering of pooled QC samples, indicating good stability of the experimental method and high data quality.

### KEGG pathway annotation of metabolites

**Figure 2:**
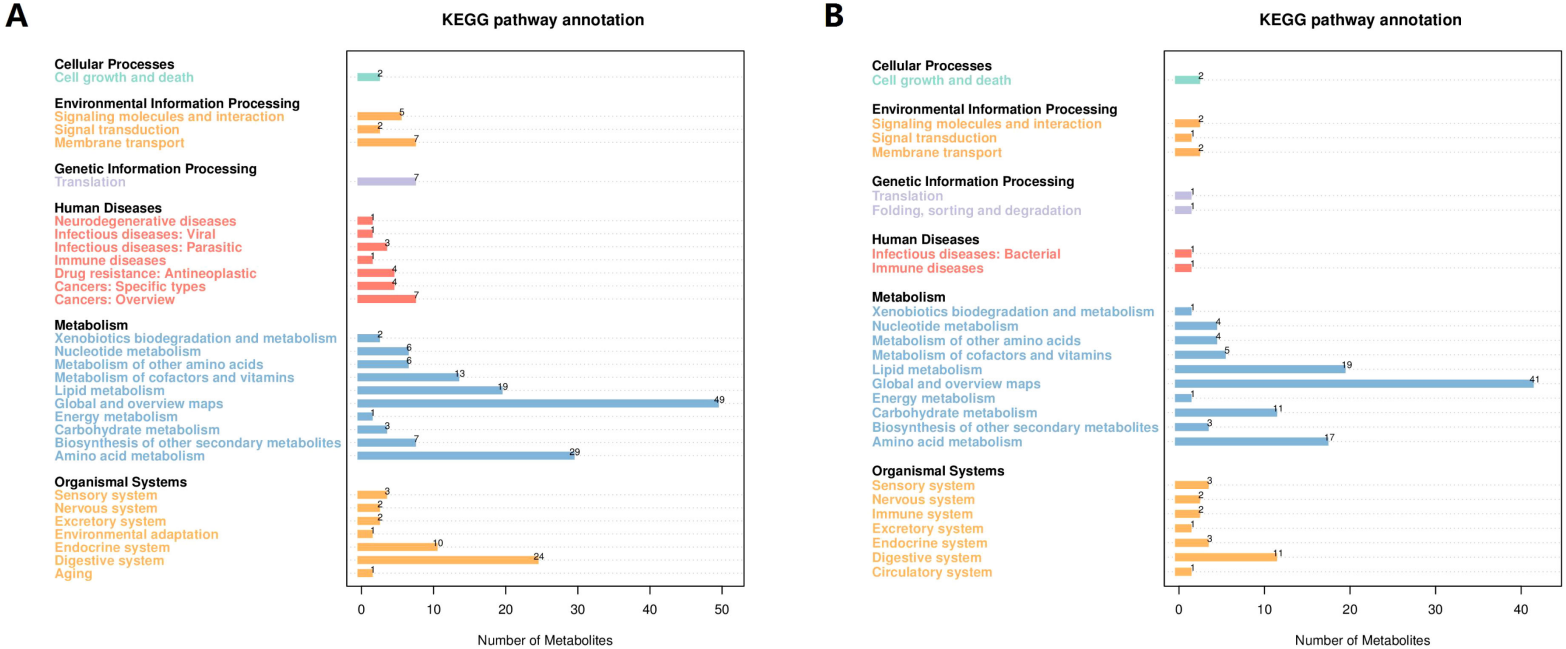
The KEGG pathway annotation of the identified metabolites. **(A)** The positive ion mode. **(B)** The negative ion mode. KEGG, Kyoto Encyclopedia of Genes and Genomes.

The KEGG pathway analysis showed that the identified metabolites were involved in metabolic pathways including cellular processes, environmental information processing, genetic information processing, human diseases, metabolism, and organic systems. The main biological metabolic pathway involved was metabolism in positive/negative ion mode (**Figures 2A, B**).

### Comparison of the E-LUAD and HC

Compared with the HC group, 82 metabolites were upregulated and eight metabolites were downregulated in the E-LUAD group in positive ion mode (**Supplementary Table 3**). In negative ion mode, 30 metabolites were upregulated and one metabolite was downregulated in the E-LUAD group **(Supplementary Table 4**).

**Figure 3:**
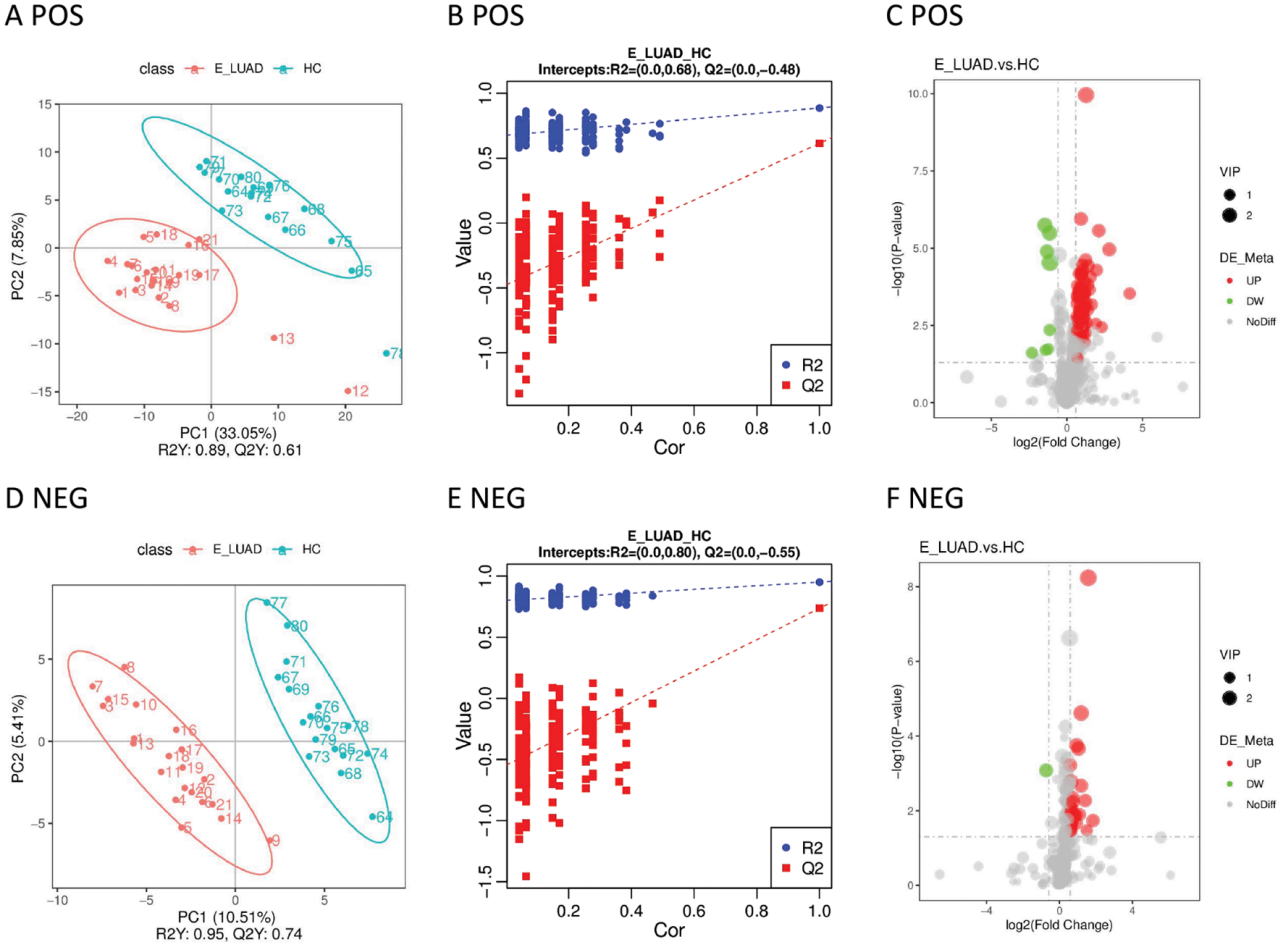
**(A)** The PLS-DA model in positive ion mode. **(B)** The permutation test in positive ion mode. **(C)** The volcano plot in positive ion mode. **(D)** The PLS-DA model in negative ion mode. **(E)** The permutation test in negative ion mode. **(F)** The volcano plot in negative ion mode. PLS-DA, partial least squares discriminant analysis.

The PLS-DA model showed that the samples in the E-LUAD and HC groups were distributed in different quadrants (**Figures 3A, D**), indicating significant metabolic differences between the two groups. The permutation test demonstrated that the PLS-DA model was not overfitting: in positive and negative modes, the Q^2^ regression line had a negative intercept, and all blue Q^2^ points were below the original blue Q^2^ point (**Figures 3B, E**). The volcano plot of the overall characteristics of the metabolite peaks is shown in **Figures 3C, F**.

The metabolic pathways are visually displayed in **Supplementary Figures 2**, **3**. The details of metabolic pathways are presented in **Supplementary Table 9**. The KEGG pathway enrichment analysis of differential metabolites showed six metabolic pathways with a p-value < 0.05 in positive ion mode, including steroid hormone biosynthesis, ovarian steroidogenesis, aldosterone synthesis and secretion, cortisol synthesis and secretion, Cushing’s syndrome, and the prolactin signaling pathway.

Plotting the ROC curves of these metabolites, the AUC values of the differential metabolites are shown in **Table 2**; **Supplementary Tables 3**, **4**. Most metabolites had a moderate discrimination ability.

**Table 2:** The AUCs of the differential metabolites (E-LUAD vs. HC).

positive ion mode		negative ion mode	
AUC	Metabolites, n (%)	AUC	Metabolites, n (%)
<0.7	1 (1.11)	<0.7	5 (16.13)
0.7–0.9	84 (93.33)	0.7–0.9	25 (80.65)
>0.9	5 (5.56)	>0.9	1 (3.23)

AUC, area under the curve; E-LUAD, early-stage lung adenocarcinoma; HC, healthy control.

### Comparison of the E-LUAD and NCC

Compared with the NCC group, there were 33 differentially expressed metabolites (25 upregulated and eight downregulated) in the E-LUAD group in positive ion mode (**Supplementary Table 5**). In negative ion mode, 33 metabolites were upregulated and one metabolite was downregulated in the E-LUAD group (**Supplementary Table 6**).

**Figure 4:**
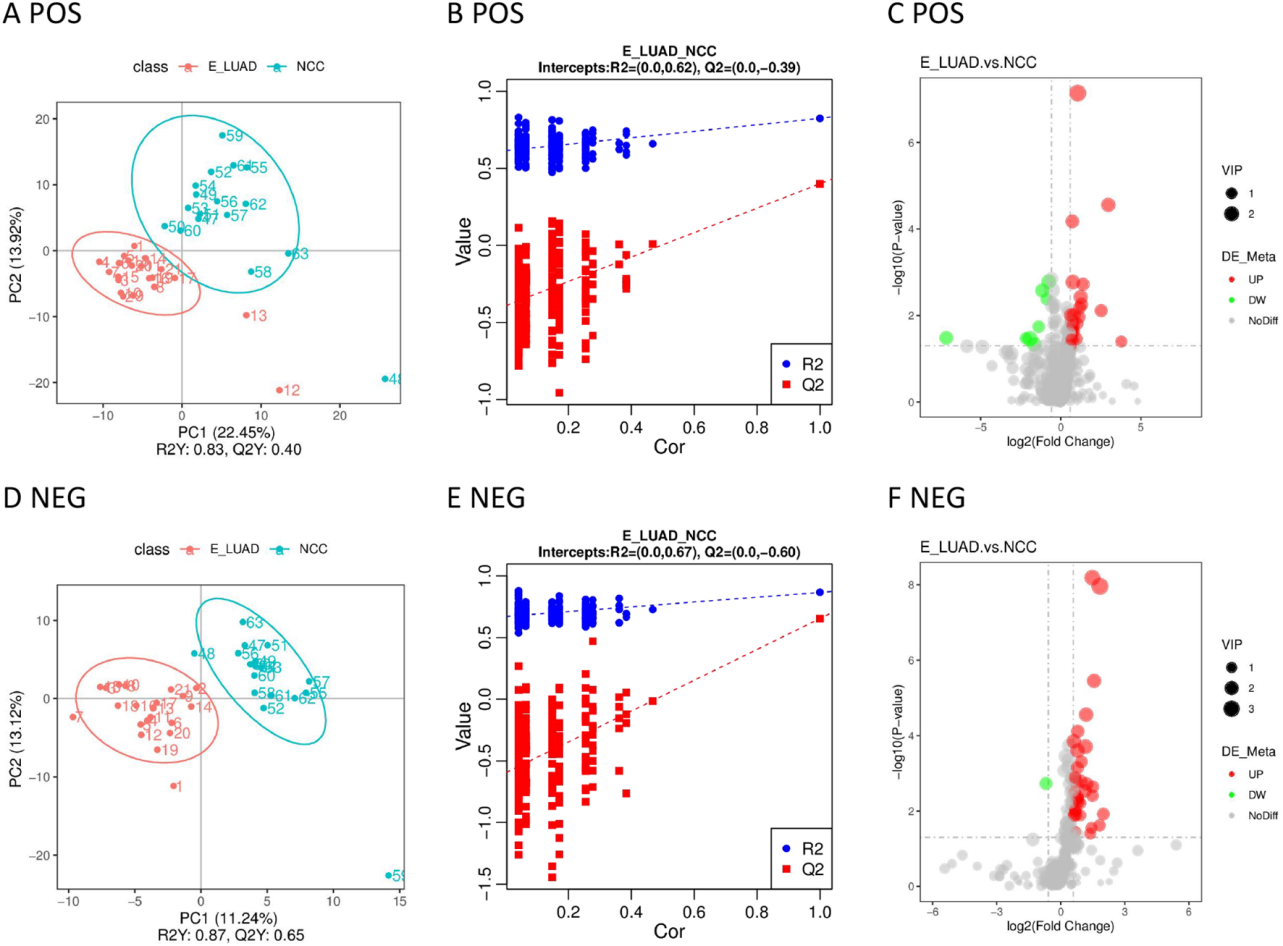
**(A)** The PLS-DA model in positive ion mode. **(B)** The permutation test in positive ion mode. **(C)** The volcano plot in positive ion mode. **(D)** The PLS-DA model in negative ion mode. **(E)** The permutation test in negative ion mode. **(F)** The volcano plot in negative ion mode. PLS-DA, partial least squares discriminant analysis.

Significant metabolic differences between the two groups were displayed in the PLS-DA model (**Figures 4A, D**). The metabolites of the two groups were distributed in different quadrants. The permutation test demonstrated that the PLS-DA model was not overfitting (**Figures 4B, E**). The volcano plot visually displayed differential metabolites between the two groups (**Figures 4C, F**).

The metabolic pathways are visually displayed in **Supplementary Figures 4**, **Supplementary Tables 5**. The details of metabolic pathways are presented in **Supplementary Table 9**. The KEGG pathway enrichment analysis of differential metabolites showed five metabolic pathways (aldosterone synthesis and secretion, cortisol synthesis and secretion, porphyrin and chlorophyll metabolism, steroid hormone biosynthesis, and Cushing’s syndrome) with a p-value < 0.05 in positive ion mode and one metabolic pathway (biosynthesis of unsaturated fatty acids) in negative ion mode.

Most differential metabolites had moderate discrimination ability (**Table 3**). Detailed information on these metabolites can be found in **Supplementary Tables 5**, **6**.

**Table 3:** The AUCs of the differential metabolites (E-LUAD vs. NCC).

positive ion mode		negative ion mode	
AUC	Metabolites, n (%)	AUC	Metabolites, n (%)
<0.7	3 (9.09)	<0.7	1 (2.94)
0.7–0.9	28 (84.85)	0.7–0.9	30 (88.24)
>0.9	2 (6.06)	>0.9	3 (8.82)

AUC, area under the curve; E-LUAD, early-stage lung adenocarcinoma; NCC, non-cancerous lung disease.

### Comparison of the E-LUAD and A-LUAD

Compared with the A-LUAD group, we identified nine metabolites with upregulated expression and 31 metabolites with downregulated expression in the E-LUAD group in positive ion mode (**Supplementary Table 7**). In negative ion mode, 13 metabolites were upregulated and one metabolite was downregulated in the E-LUAD group (**Supplementary Table 8**).

**Figure 5:**
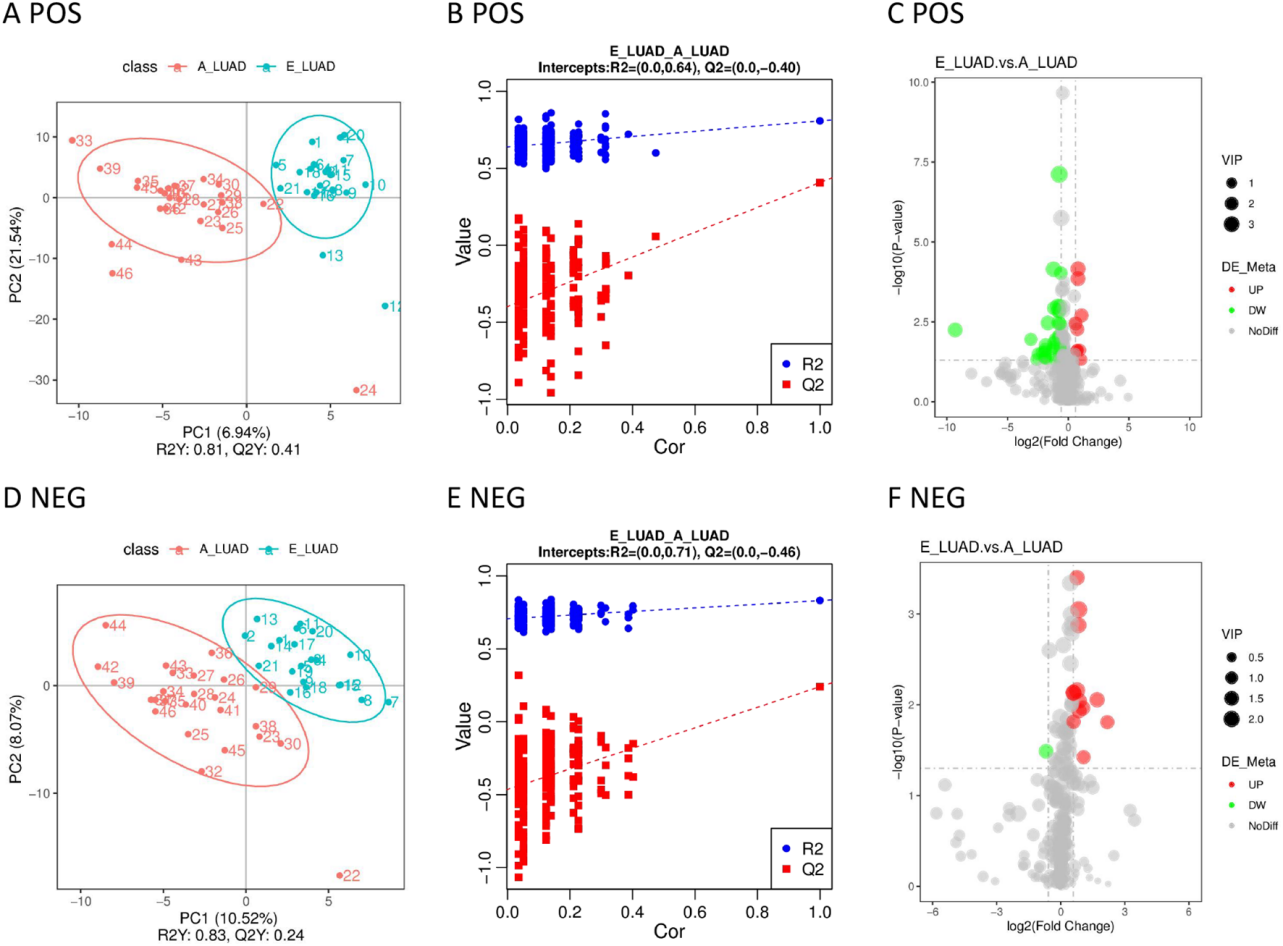
**(A)** The PLS-DA model in positive ion mode. **(B)** The permutation test in positive ion mode. **(C)** The volcano plot in positive ion mode. **(D)** The PLS-DA model in negative ion mode. **(E)** The permutation test in negative ion mode. **(F)** The volcano plot in negative ion mode. PLS-DA, partial least squares discriminant analysis.

The samples distributed in different quadrants in the PLS-DA model indicated the significant metabolic differences between the E-LUAD and A-LUAD (**Figures 5A, D**). The permutation test demonstrated that the PLS-DA model was not overfitting (**Figures 5B, E**). The volcano plot visually displayed the overall characteristics of the metabolites (**Figures 5C, F**).

The metabolic pathways are visually displayed in **Supplementary Figures 6**, **7**. The details of metabolic pathways are presented in **Supplementary Table 9**. The KEGG pathway enrichment analysis of differential metabolites showed only one metabolic pathway (bile secretion) with a p-value < 0.05 in positive ion mode.

The AUCs of 87.5% (35/40) of the metabolites were greater than 0.70 in positive ion mode, but only one (2.5%) metabolite had an AUC greater than 0.9 (**Table 4**; **Supplementary Table 7**). The AUCs of 71.43% (10/14) of the metabolites were greater than 0.70 in negative ion mode; however, none of the metabolites had an AUC greater than 0.9 (**Table 4**; **Supplementary Table 8**).

**Table 4:** The AUCs of the differential metabolites (E-LUAD vs. A-LUAD).

Positive ion mode		Negative ion mode	
AUC	Metabolites, n (%)	AUC	Metabolites, n (%)
<0.7	5 (12.5)	<0.7	4 (28.57)
0.7–0.9	34 (85)	0.7–0.9	10 (71.43)
>0.9	1 (2.5)	>0.9	0 (0)

AUC, area under the curve; E-LUAD, early-stage lung adenocarcinoma; A-LUAD, advanced lung adenocarcinoma.

### Specific biomarker identification

The heatmap based on the differentially expressed metabolites provided intuitive visualizations of the trends in metabolites between the four groups in positive/negative ion mode (**Supplementary Figures 8**, **9**). As can be seen intuitively from the heatmap, different metabolites increased or decreased in samples of the four groups.

**Figure 6:**
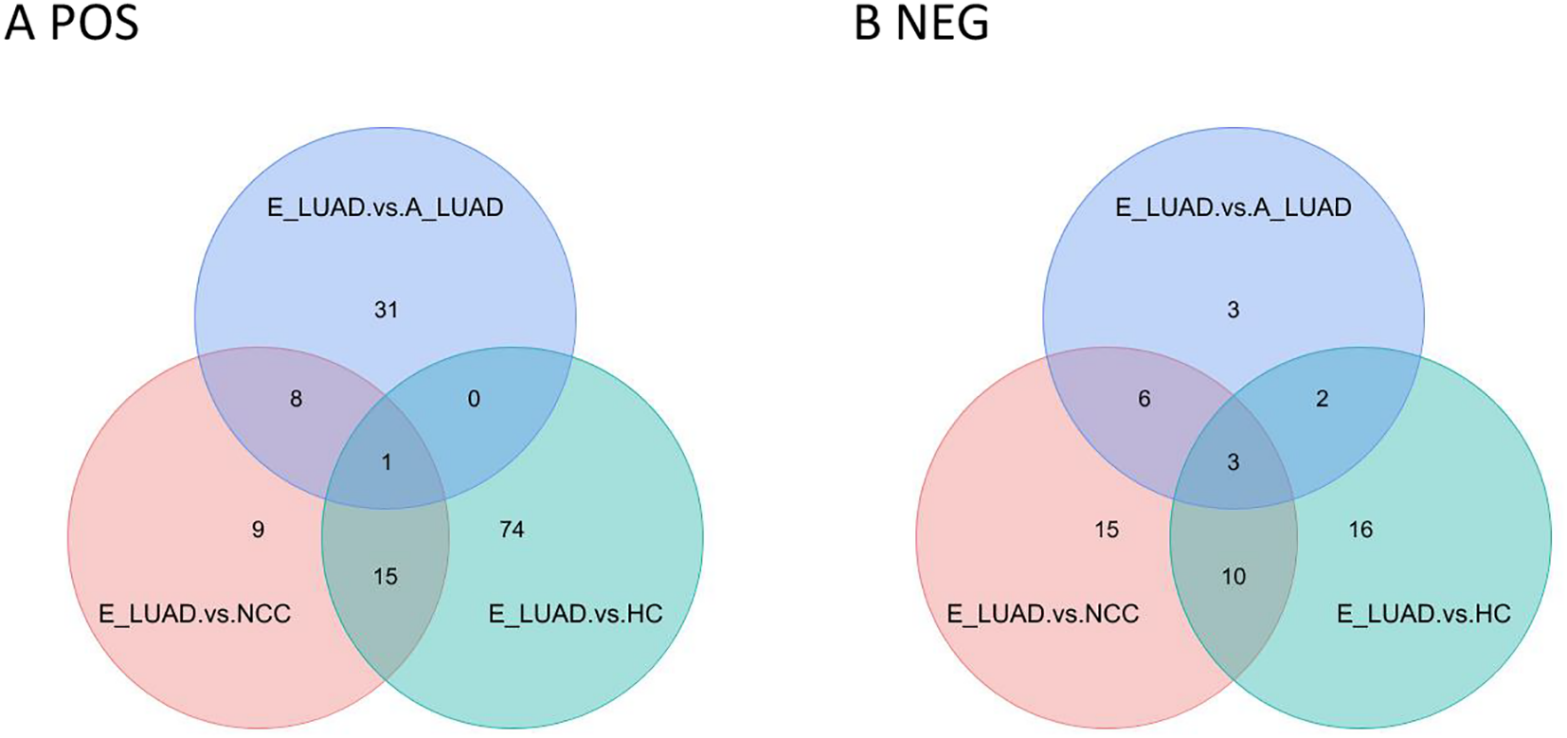
Venn diagram of the three comparisons. **(A)** The positive ion mode. **(B)** The negative ion mode.

The Venn diagram visualized the overlapping results between the differentially regulated metabolites found at E-LUAD vs. HC, E-LUAD vs. NCC, and E-LUAD vs. A-LUAD comparisons (**Figure 6**).

In positive ion mode, 16 metabolites were commonly differentially regulated between E-LUAD vs. HC and E-LUAD vs. NCC comparisons, indicating that 16 metabolites can simultaneously distinguish E-LUAD from HC and NCC; 81.25% of these 16 metabolites had AUCs between 0.7 and 0.9, and 12.5% had AUCs greater than 0.9. In negative ion mode, the Venn diagram visualized 13 overlapping differential metabolites between E-LUAD vs. HC and E-LUAD vs. NCC comparisons, indicating that 13 metabolites can simultaneously distinguish E-LUAD from HC and NCC; 76.92% of these 13 metabolites had AUCs between 0.7 and 0.9, and 7.69% had AUCs greater than 0.9.

In positive ion mode, only one metabolite was commonly differentially regulated between E-LUAD vs. HC, E-LUAD vs. NCC, and E-LUAD vs. A-LUAD comparisons, indicating that only one metabolite can simultaneously distinguish E-LUAD from HC, NCC, and E-LUAD. In negative ion mode, the Venn diagram visualized three overlapping differential metabolites, indicating that only three metabolites were specific diagnostic markers for E-LUAD.

**Figure 7:**
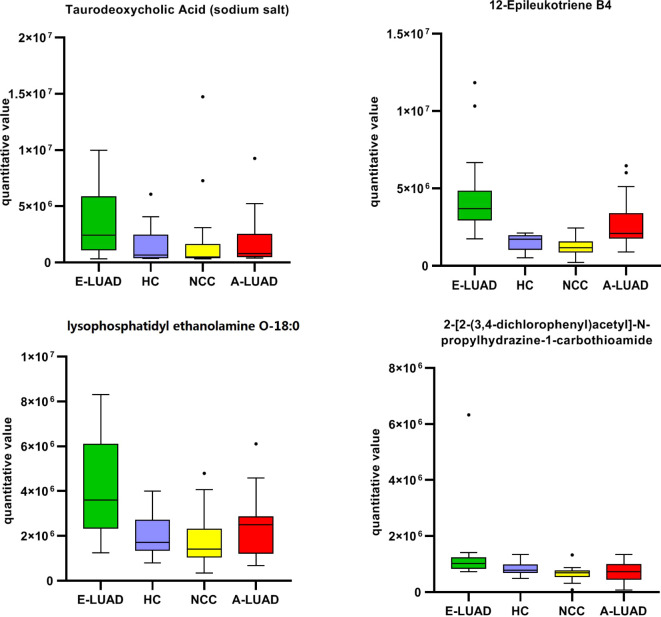
The box plot of the commonly differentially regulated metabolites between E-LUAD vs. HC, E-LUAD vs. NCC, and E-LUAD vs. A-LUAD comparisons. E-LUAD, early-stage lung adenocarcinoma; HC, healthy control; NCC, non-cancerous lung disease; A-LUAD, advanced lung adenocarcinoma.

Compared with the other three groups, the box plot showed that the four metabolites were significantly upregulated in the E-LUAD group (**Figure 7**, colored green).

The AUCs of the four metabolites are shown in **Table 5**. The AUC of one ROC curve was less than 0.70 (0.690), and the AUCs of the remaining ROC curves were all greater than 0.70. The detailed information on these four metabolites is presented in **Table 6**.

**Table 5:** The AUCs of the commonly differentially regulated metabolites.

Metabolites	E-LUAD vs. HC	E-LUAD vs. NCC	E-LUAD vs. A-LUAD
Taurodeoxycholic acid (sodium salt)	0.725	0.754	0.690
12-Epileukotriene B4	0.978	0.992	0.792
Lysophosphatidylethanolamine O-18:0	0.818	0.832	0.777
2-[2-(3,4-dichlorophenyl)acetyl]-*N*-propylhydrazine-1-carbothioamide	0.754	0.899	0.790

E-LUAD, early-stage lung adenocarcinoma; HC, healthy control; NCC, non-cancerous lung disease; A-LUAD, advanced lung adenocarcinoma.

**Table 6:** Detailed information on metabolites.

Name	Formula	Molecular weight	retention time (RT)	m/z
Taurodeoxycholic acid (sodium salt)	C_26_H_45_NO_6_S	499.29714	7.706	500.30441
12-Epileukotriene B4	C_20_H_32_O_4_	336.23033	7.347	335.22305
Lysophosphatidylethanolamine (LPE) O-18:0	C_23_H_50_NO_6_P	467.33798	10.91	466.33071
2-[2-(3,4-Dichlorophenyl)acetyl]-*N*-propylhydrazine-1-carbothioamide	C_12_H_15_Cl_2_N_3_OS	355.00429	6.129	353.99701

### Diagnostic model construction for E-LUAD

The data were randomly divided into a training set and a validation set in a 7:3 ratio. The Venn diagram showed that four metabolites can distinguish E-LUAD from HC, NCC, and E-LUAD. Logistic regression analysis showed that except for taurodeoxycholic acid (sodium salt), the p-values of the other three metabolites were all less than 0.05. The three identified metabolites, namely, 12-epileukotriene B4, LPE O-18:0, and 2-[2-(3,4-dichlorophenyl)acetyl]-*N*-propylhydrazine-1-carbothioamide, were used as diagnostic feature biomarkers for predicting E-LUAD and constructing a diagnostic model.

**Figure 8:**
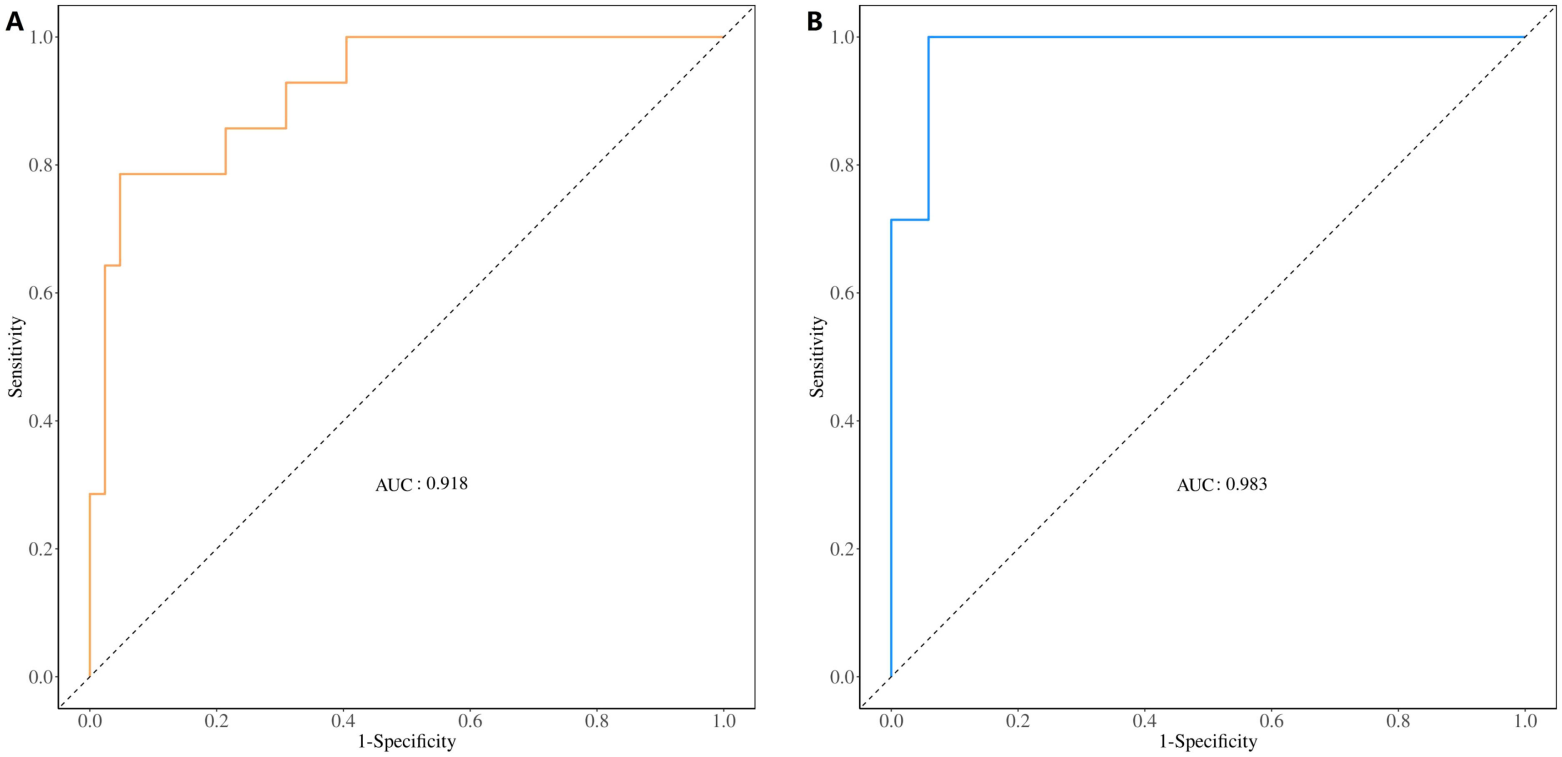
ROC curve for evaluating and validating the diagnostic model’s performance. **(A)** ROC curve of the training set. **(B)** ROC curve of the validation set. ROC, receiver operating characteristic.

The ROC curve was used to evaluate the diagnostic performance of the model. The AUC value of the training set was 0.918 (**Figure 8A**), and it was 0.983 in the validation set (**Figure 8B**), indicating that the diagnostic model exhibited a good predictive value for E-LUAD.

## Discussion

Various types of metabolomic samples are available for the diagnosis of lung cancer. The first choice for screening early lung cancer is a non-invasive examination. The concentration of urine varies greatly throughout the day, and the preservation of breath samples is difficult. We believe that blood is more suitable as an early diagnostic sample for lung cancer. Blood metabolomics has been widely applied in the study of disease biomarkers, and it is one of the most common sample types in metabolomics research (5). In our study, we collected plasma for an untargeted metabolomics study.

The levels of many metabolites in the human plasma metabolome are influenced by many factors such as sex, age, smoking, the composition of their diet, gut microbiome, an individual’s genetics, collection tube, and underlying diseases (6–8). Given that metabolomics is influenced by many factors, controlling non-experimental factors is crucial for the stability and accuracy of experimental results. In theory, all diseases may have their own unique metabolomic features, and any diseases that patients suffer from may cause varying degrees of changes in blood metabolomics. At present, most studies on lung cancer metabolomics only use healthy individuals as the control group (9–18). The lack of benign lung diseases in the control group may result in differential metabolites screened in the study not being specific markers for lung cancer, as these metabolites may also exhibit similar changes in benign diseases compared to healthy controls. The control group of a few studies included healthy individuals and non-cancerous lung diseases (19, 20). For early-stage lung cancer, we believe that these studies also have some shortcomings. The markers screened using this method can only indicate that they are markers for lung cancer but may not necessarily be markers for early-stage lung cancer. Due to the lack of comparison between early-stage and advanced lung cancer, these metabolic markers may have similar changes in early-stage and advanced lung cancer, making it difficult to distinguish between them. Our research identified numerous lung cancer metabolic markers that align with previous studies, such as upregulated hexanoylcarnitine, cortisol, cholic acid, lithocholic acid, tetradecanedioic acid, and lysophosphatidic acid (LPA) 18:2 (4, 10, 21). However, there is significant heterogeneity in metabolomics research. Many metabolites, such as choline, xanthine, hypoxanthine, linoleic acid, stearic acid, oleic acid, citrulline, arginine, tryptophan, and ornithine, have been reported as upregulated in lung cancer in some studies, while other studies have shown the opposite trend (4). The reasons for these discrepancies between different studies are unclear and may be due to subject selection criteria or patient heterogeneity. Similar inconsistencies exist between our study and previous research. For example, lysophosphatidylcholine (LPC) 18:0 was downregulated in our study but upregulated in another study (10).

In our study, participants were divided into four groups: E-LUAD, HC, NCC, and A-LUAD. The E-LUAD group was compared with the other three groups to analyze differential metabolites. Then, a Venn diagram was constructed to identify overlapping results between the differentially regulated metabolites found at E-LUAD vs. HC, E-LUAD vs. NCC, and E-LUAD vs. A-LUAD comparisons. From the Venn diagram, 29 differentially regulated metabolites were identified between E-LUAD and the two non-cancerous control groups, indicating that the 29 metabolite markers can distinguish E-LUAD from healthy individuals and non-cancerous lung diseases. Four differentially regulated metabolites were identified between E-LUAD and the other three groups, indicating that these metabolites can additionally distinguish early stage from advanced lung adenocarcinoma. A diagnostic model was constructed for E-LUAD using the identified metabolites, and the ROC curve showed that the model exhibited a good predictive value (AUC > 0.9). In addition, in our study, 121 differential metabolites were identified by comparing E-LUAD with HC, while only 67 differential metabolites were identified by comparing E-LUAD with NCC. Compared with A-LUAD, the lowest number (only 54) of differential metabolites was identified, indicating that the difficulty of distinguishing E-LUAD from HC, NCC, and A-LUAD is gradually increasing.

In our study, 2-[2-(3,4-dichlorophenyl)acetyl]-*N*-propylhydrazine-1-carbothioamide has potential diagnostic value for E-LUAD. According to the Chemical Entities of Biological Interest (ChEBI) database, the species of this metabolite is *Homo sapiens*. Its biological function is unclear. This metabolite can be found in peripheral blood mononuclear cells. It is a dichlorobenzene, which may be a potential carcinogen. Therefore, it is possible that this metabolite is derived from an exogenous source/manufacturer. As cancer progresses, the concentration of this metabolite may decrease, which may be the reason why it can distinguish between E-LUAD and A-LUAD.

12-Epileukotriene B4 is a non-enzymatically derived isomer of leukotriene B4 (22). Leukotriene B4 was found to induce epithelial–mesenchymal transition in human adenocarcinoma alveolar basal epithelial A549 cells (23). It may play an important role in lung cancer progression. Leukotriene B4 was upregulated in the lungs of the mice with lung cancer (24). Compared to that in healthy smoking/non-smoking controls, leukotriene B4 resulted higher in breath condensate in lung cancer patients (25, 26). In our study, leukotriene B4 was upregulated in the blood and has potential diagnostic value for early lung cancer.

As a breakdown product of phosphatidylethanolamine (PE), lysophosphatidylethanolamine (LPE) is present in the cells of all organisms. Mass spectrometry-based studies have demonstrated that LPE serves as a prognostic marker in cancer (27). Lei et al. (28) performed metabolomics analysis in 131 patients with their lung tissue pairs. Compared with paired distal non-cancerous tissues, LPE 16:0 significantly increased in lung carcinoma tissues. Noreldeen et al. (29) observed that the levels of serum LPE increased in the non-smoking female patients with non-small cell lung cancer, compared with the healthy controls. Serum LPE (20:4) can serve as a biomarker for distinguishing female patients with non-small cell lung cancer from healthy controls. Our study demonstrated that plasma LPE O-18:0 can distinguish E-LUAD from HC, NCC, and A-LUAD.

Tang et al. (3) reported that taurodeoxycholic acid 3-sulfate was positively associated with lung cancer risk. The association of taurodeoxycholic acid 3-sulfate with lung cancer was the strongest among cases diagnosed within 3 years of follow-up. It is a potential screening biomarker for lung cancer. Although not as good as the other three metabolites, taurodeoxycholic acid (sodium salt) still has a certain differential diagnostic value in our study.

In recent years, an increasing amount of metabolites has been detected, but many metabolites have not been reflected in a better understanding of metabolic pathways. Most of the metabolic pathway differences were in hormone biosynthesis pathways in our study, particularly steroid hormones. Steroid hormones are involved in the biology of lung cancer, but to what extent they contribute to lung cancer is unclear (30). The lung contains receptors for both estrogen and progesterone. Several studies indicated the possible involvement of sex steroids in both the development and progression of lung cancer (31). Bile secretion was a different metabolic pathway for early- vs. late-stage lung cancer in our study. Long et al. (32) reported that total bile acids had the potential to distinguish between advanced and early-stage lung cancer. Some of the studies have characterized the role of bile acids in cancer development and progression (33). However, details remain unclear on how bile acid metabolism is regulated in lung cancer.

Our research also has shortcomings. First, the sample size was small. Second, we did not conduct targeted metabolomics validation. With the improvement of detection instrument performance, if conditions permit, large-scale metabolomics studies can be conducted in the future to minimize the influence of non-experimental factors. It is promising to screen out metabolic biomarkers specific for early-stage lung cancer that are applicable to clinical practice. Nonetheless, differential metabolites are not equal to disease biomarkers; the application of metabolomics in clinical practice still has a long way to go.

The future direction of metabolomics research is to identify reliable biomarkers that can help in distinguishing between lung cancer and non-cancerous lung diseases, and various lung cancer types and stages. Targeted metabolomics studies will be necessary to verify the reliability of findings from non-targeted metabolomics. Metabolomics signatures must first be validated on larger cohorts, followed by the implementation of quantitatively robust methods for the metabolites of interest. Finally, prospective clinical trials must be conducted to validate the reliability and adaptability of the biomarkers. With technological advancement and the decrease in testing costs, metabolomics may be able to replace more costly and invasive diagnostic procedures and provide easy and cost-effective methods for first-line diagnosis.

## Conclusions

Blood metabolomics has potential diagnostic value for E-LUAD. More medical studies are needed to verify whether the metabolic markers identified in current research can be applied in clinical practice.

## Data Availability

The datasets presented in this study are deposited in online repositories. The names of the repositories and accession numbers can be found below: https://data.mendeley.com/datasets/cv6x58scpw/1; https://data.mendeley.com/datasets/zk2k6tnknt/1; https://data.mendeley.com/datasets/pg4z7jwwr7/1.
