# Supplementary figures and images for: Metabolomics for early-stage lung adenocarcinoma: diagnostic biomarker screening
Source: Front Oncol. 2025 Mar 11;15:1535525. doi: 10.3389/fonc.2025.1535525 (PMC11932905; doi:10.3389/fonc.2025.1535525)

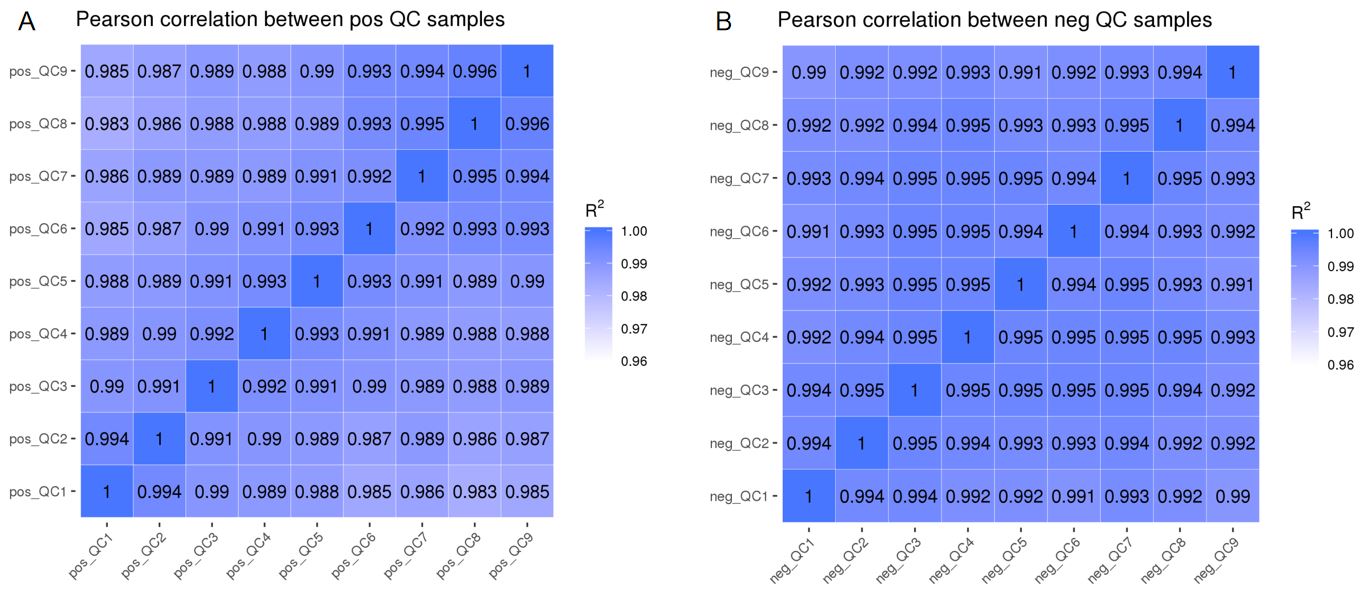

Supplement: Supplementary file 1 [file DataSheet1.zip › Supplementary Figure 1.JPG]

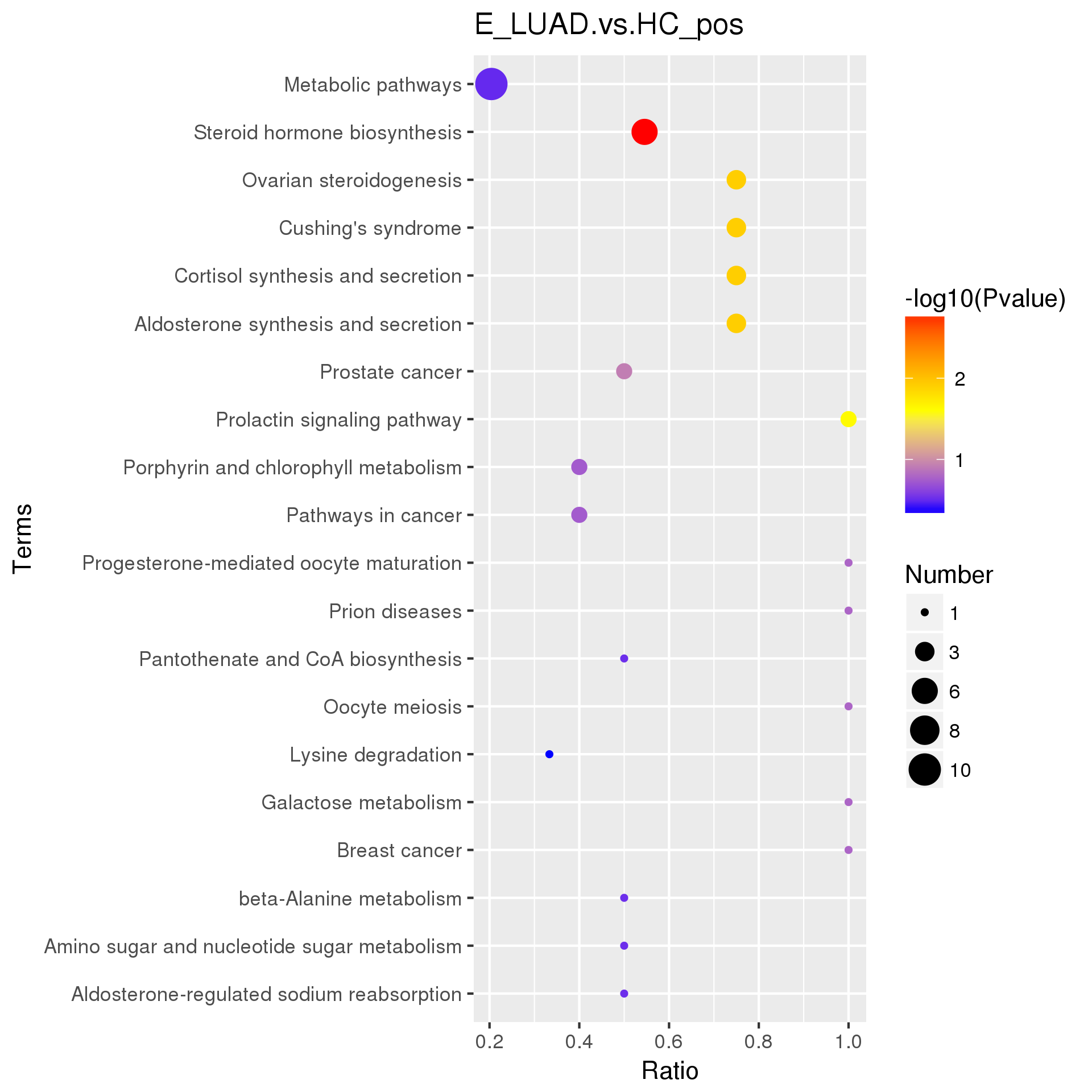

Supplement: Supplementary file 1 [file DataSheet1.zip › Supplementary Figure 2.png]

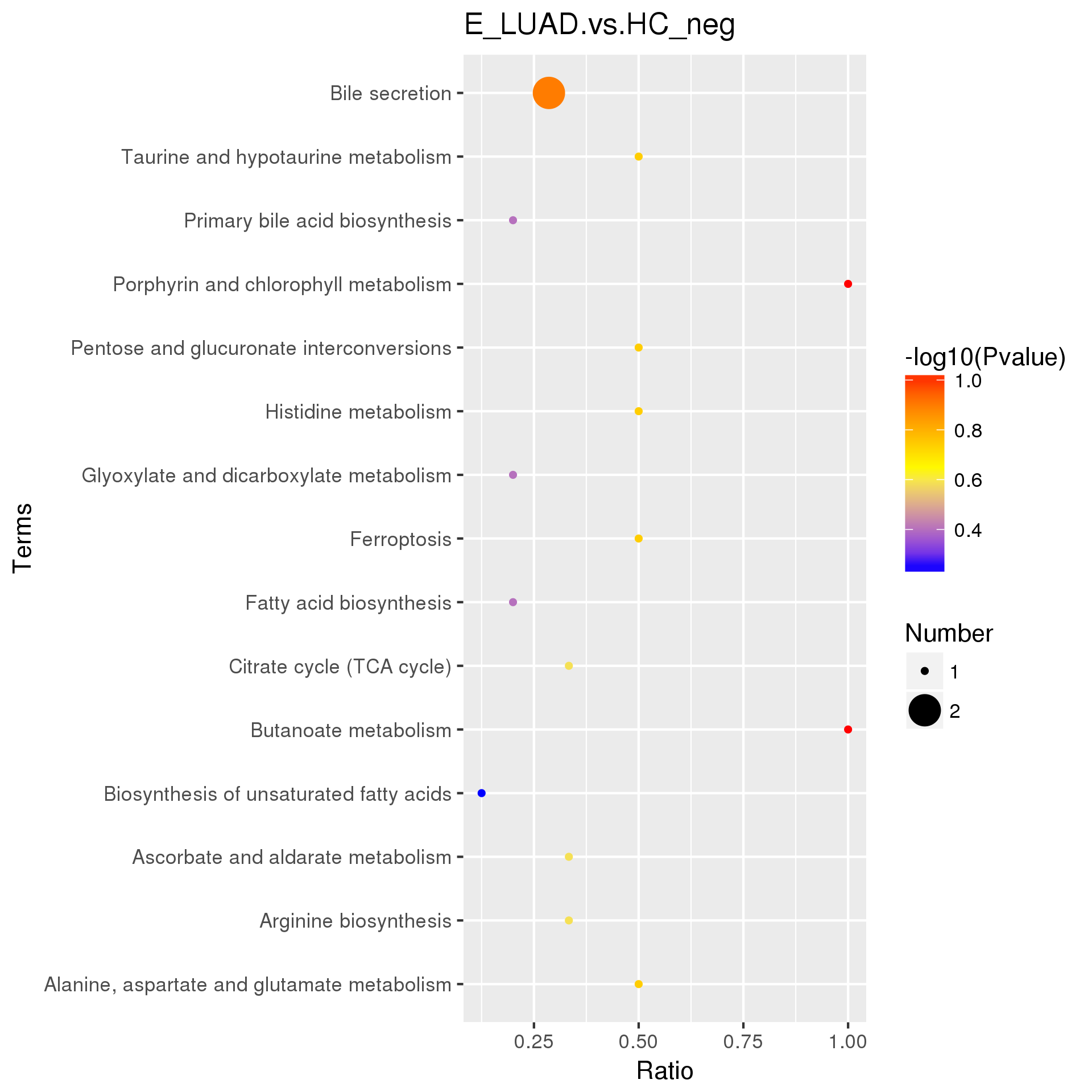

Supplement: Supplementary file 1 [file DataSheet1.zip › Supplementary Figure 3.png]

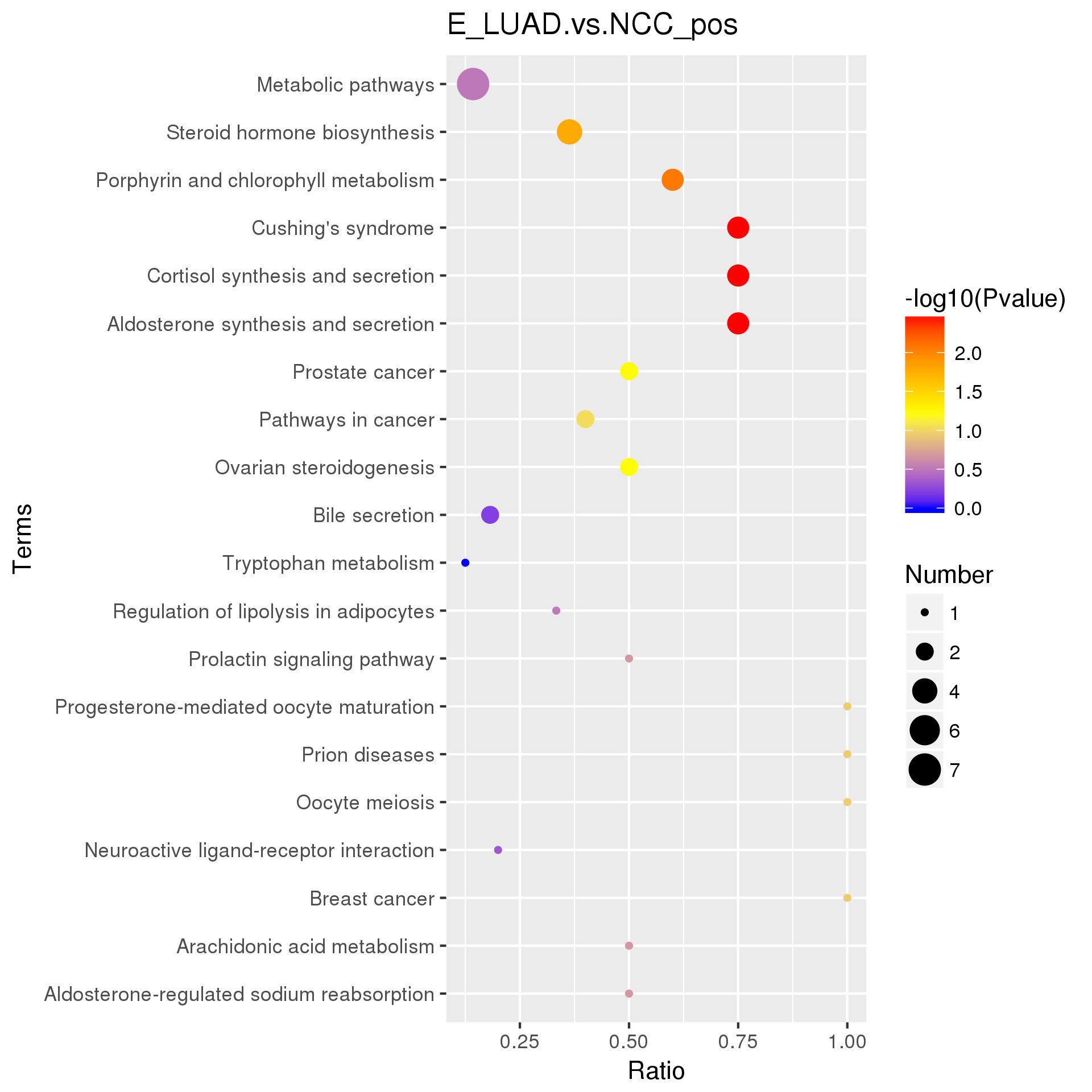

Supplement: Supplementary file 1 [file DataSheet1.zip › Supplementary Figure 4.png]

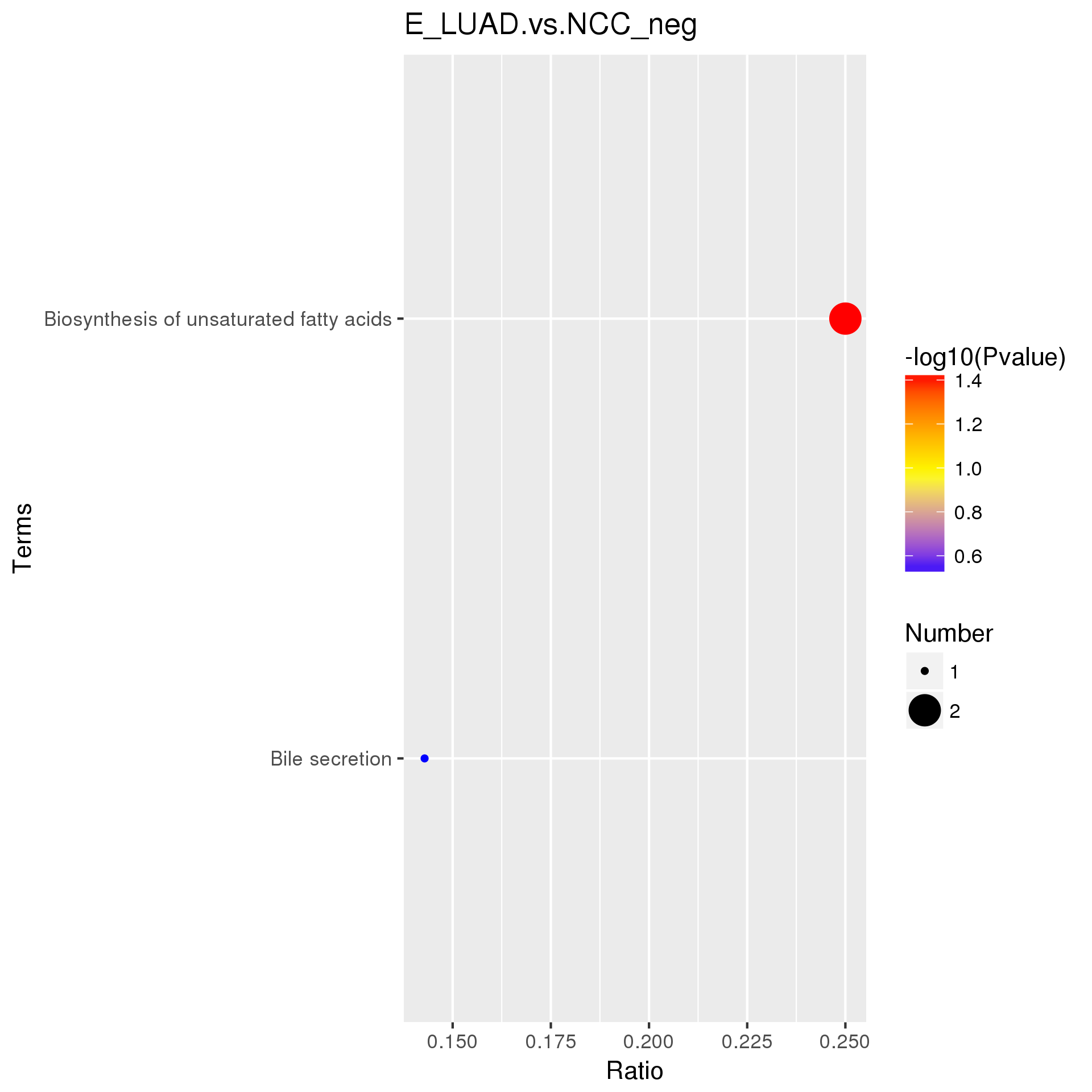

Supplement: Supplementary file 1 [file DataSheet1.zip › Supplementary Figure 5.png]

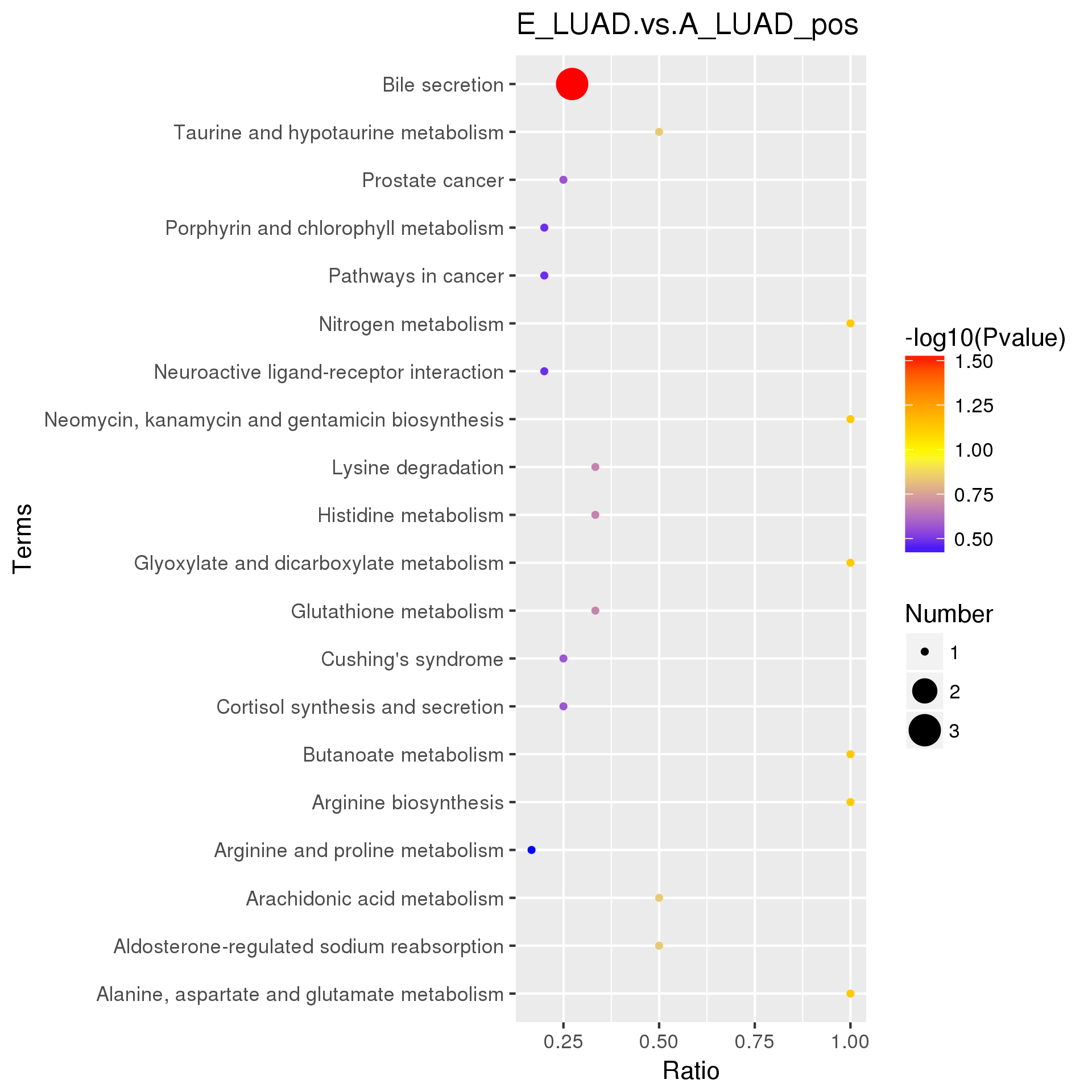

Supplement: Supplementary file 1 [file DataSheet1.zip › Supplementary Figure 6.png]

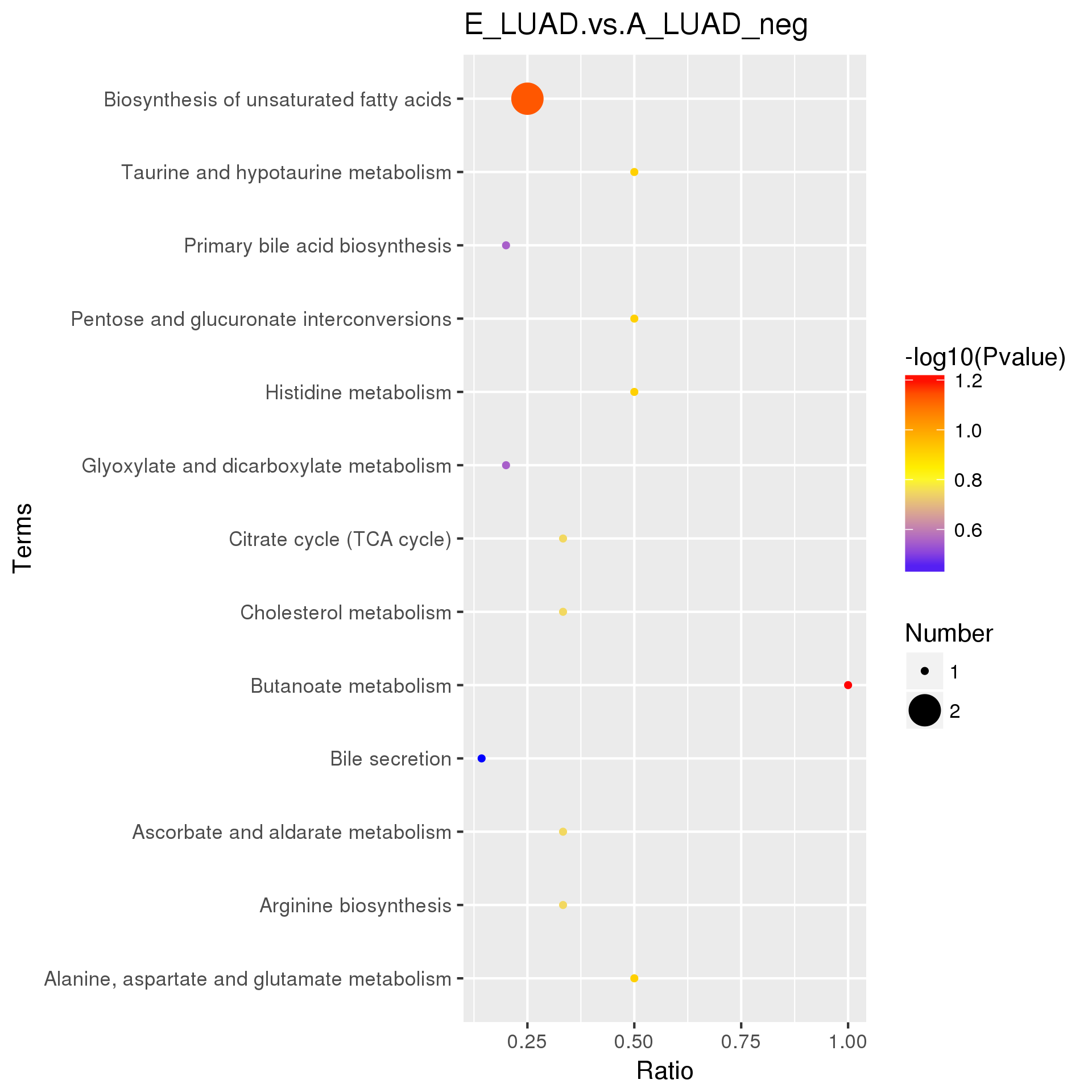

Supplement: Supplementary file 1 [file DataSheet1.zip › Supplementary Figure 7.png]

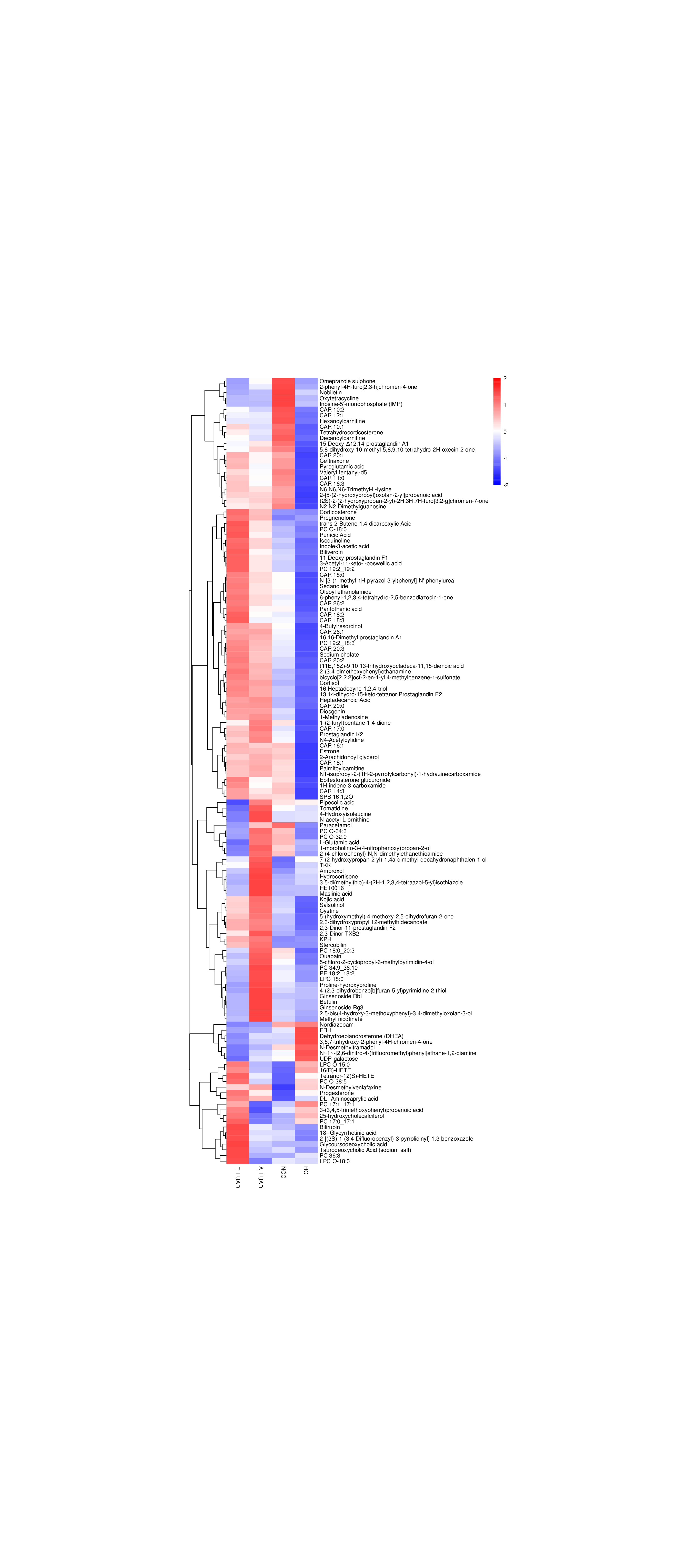

Supplement: Supplementary file 1 [file DataSheet1.zip › Supplementary Figure 8.png]

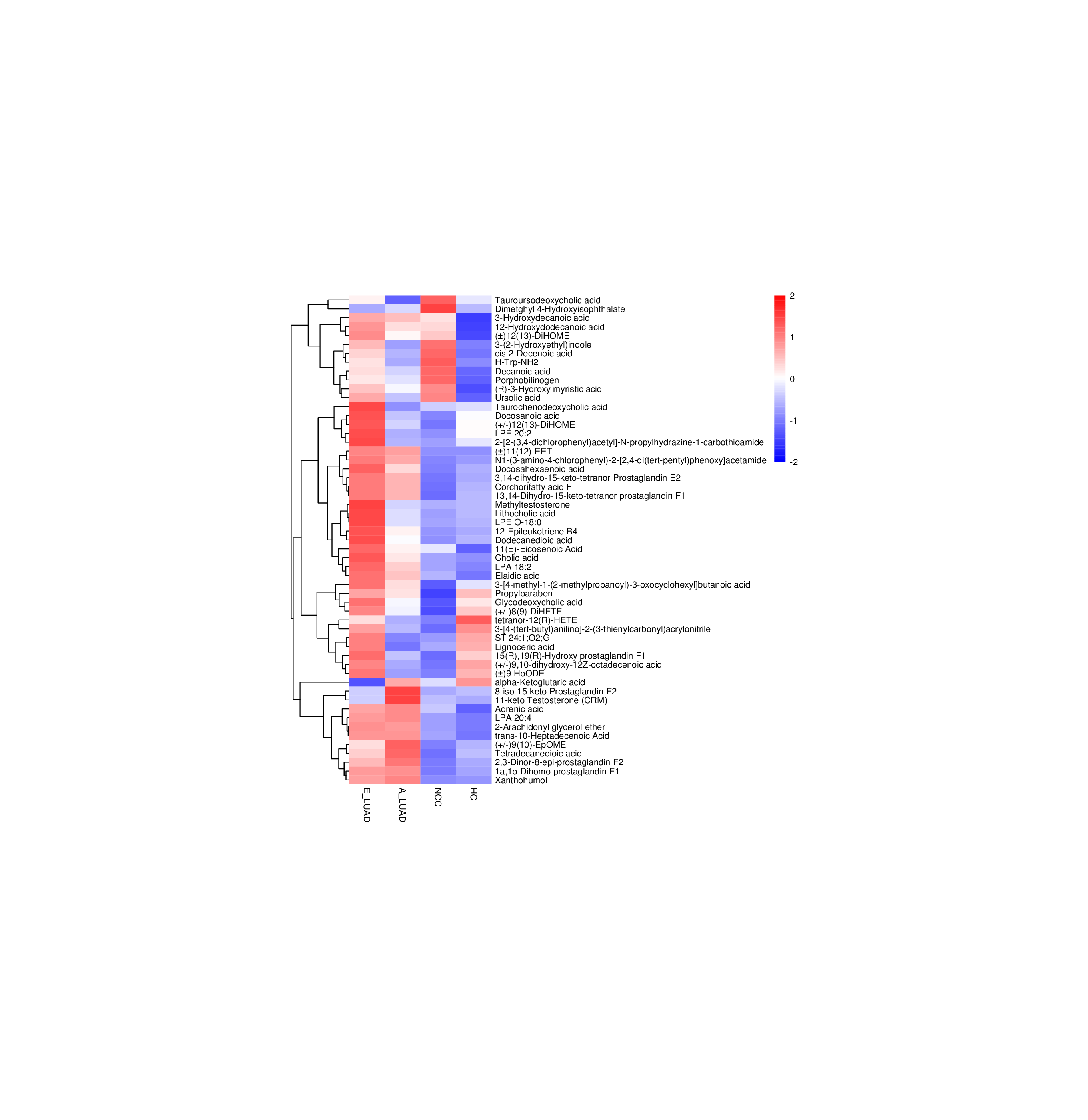

Supplement: Supplementary file 1 [file DataSheet1.zip › Supplementary Figure 9.png]
